# A Case of Medialized Lateral Maxillary Sinus Wall: A Pillar of Support

**DOI:** 10.1155/2018/4053531

**Published:** 2018-05-13

**Authors:** I. A. Othman, N. D. Hashim, A. J. Nazimi

**Affiliations:** ^1^Department of Otorhinolaryngology, Head & Neck Surgery, Faculty of Medicine, Universiti Kebangsaan Malaysia, Cheras, Kuala Lumpur, Malaysia; ^2^Department of Otorhinolaryngology, Head & Neck Surgery, Kulliyah of Medicine, International Islamic University Malaysia, Kuantan, Pahang, Malaysia; ^3^Oral & Maxillofacial Surgery Clinic, Universiti Kebangsaan Malaysia Hospital, 56000 Kuala Lumpur, Malaysia

## Abstract

The number of maxillofacial trauma (MFT) cases attended in the Emergency Department is progressively increasing in trend, owing to the rising statistics of motor-vehicle accidents (MVAs) and urban assaults in addition to occupational-related injuries. Prompt and thorough assessment is important for accurate diagnosis and paramount treatment plans. We will be discussing a case of unusual presentation of an orbital floor fracture post-MVA which was treated conservatively based on the clinical assessments during follow-ups, supported by radiological findings. We will also briefly discuss the different radiological modalities available in assessing MFT and late presentation of enophthalmos.

## 1. Introduction

In developing countries, the growing frequency of maxillofacial trauma (MFT) cases is directly proportional to the rising numbers of motor-vehicle accidents (MVAs) reported and the increasing incidents of urban assaults [1–3]. Thorough and effective assessments, both clinical and radiological, are deemed necessary to ensure that accurate diagnosis is being made at initial presentation to avoid long-term physical impairment affecting function. Computed tomography (CT) has become the standard imaging tool in assessing MFT and is usually carried out during the first encounter in the Emergency Department (ED). Clinical examination has its limitation due to the presence of soft tissue swelling, making radiological assessment superior in detecting facial fractures. In our center, patients presented to ED with MFT with no neurological deficits are usually reassessed in oral and maxillofacial surgery (OMFS) outpatient clinic. Frequently, these patients were subjected to cone-beam CT (CBCT) or multidetector CT (MDCT) with 3D reconstruction for a more comprehensive preoperative assessment of the facial fractures. We report a case of a 33-year-old man who sustained a blowout orbital fracture with no ophthalmic abnormalities apart from periorbital hematoma, with emphasis given towards detailed imaging analysis. Various imaging modalities used in diagnosing MFTs are also briefly discussed.

## 2. Case Report

**Figure 1:**
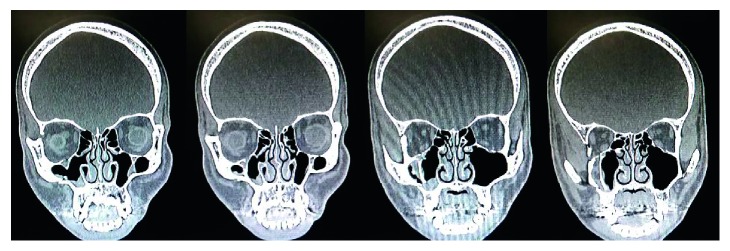
The medialized lateral wall of right maxillary sinus supports the fractured right orbital floor, maintaining the volume of the right orbit.

**Figure 2:**
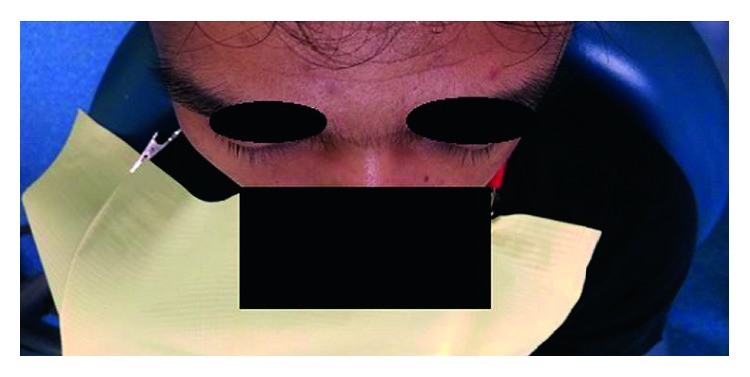
Bird's eye view showed the absence of enophthalmos.

A 33-year-old gentleman who was allegedly involved in a motor-vehicle accident was referred to our unit for further management of the suspected orbital fracture. Initial assessment in the Emergency Department (ED) showed that the patient was drowsy, not opening his eyes, or responding to questions, possibly due to alcohol intoxication. His conscious level improved gradually within 24 hours. Clinical assessment showed a right periorbital hematoma and markedly swollen right cheek. Step deformity both over the right supra- and infraorbital rim cannot be appreciated due to the swelling. Satisfactory mouth opening was also recorded. We reviewed the computed tomography (CT) done for the assessment of concomitant traumatic brain injury. The right temporal bone fracture with a fractured fragment in the temporomandibular joint (TMJ) space, the right zygomatic arch fracture, the right greater wing of the sphenoid fracture, the orbital floor fracture, and lateral and medial walls of the right maxillary sinus fracture were reported. The patient was subsequently observed in the neurosurgical ward for 48 hours prior to his discharge. Outpatient assessment in the oral and maxillofacial clinic a week after trauma noted grossly symmetrical malar prominence, with resolving right periorbital hematoma. No enophthalmos, diplopia, and eye motility restriction were clinically observed (Figure 2). Mouth opening was, however, quite limited to 25 mm interincisal. Further ophthalmology assessment confirmed good eye movement with no diplopia. The patient continued to be reviewed weekly under maxillofacial follow-up for assessment of late enophthalmos. Cone-beam CT (CBCT) orbit carried out 3 weeks after trauma confirmed findings of the previous scan. The fractured lateral wall and the floor of the right orbit (involving the infraorbital foramen), the fractured right greater wing of the sphenoid, the lateral wall of right maxillary sinus involving the right alveolar process, and the undisplaced fracture of the right zygomatic arch were observed. Detailed radiographic analysis showed that although the fracture size is small involving less than 50% of the overall size of the orbital floor, some degree of herniation albeit without periorbital entrapment was observed (Figure 1). Additionally, the orbital floor fracture occurred just immediately behind the equator of the globe, that is, at the main bulk of the inferior rectus muscle. However, its muscle height-to-width ratio remained intact. These detailed radiographic findings may suggest that the patient could develop late orbital fracture complications such as late enophthalmos, diplopia, and restriction in eye movements. These complications could occur secondary to the incarceration of periorbital tissues or muscle or later cicatrization in and around the inferior rectus muscle.

However, it was also noticeable to us that the fractured lateral wall of maxillary sinus was displaced medially along with some lateral soft tissue components. All muscles especially the lateral and inferior extraocular muscles appeared normal. Close follow-up was advocated in this case. At one month after trauma, the patient's eye movements remained intact, and there were no diplopia and apparent enophthalmos. Given these clinical findings, he was subjected to conservative treatment with regular follow-ups. The interesting component of this case is that, despite the multitude of bony fractures, the patient did not develop enophthalmos, diplopia, or restricted eye movements. Upon close review of the CBCT of the right orbit, we believe that the medialization of the fractured lateral wall of maxillary sinus has somehow acted and has healed as a pillar, partly or wholly to maintain the integrity of the right orbital floor.

## 3. Discussion

The facial skeleton can be pictured as four transverse buttresses and four paired vertical buttresses that support both the form and function of the face [4]. Any forces directed towards the facial region will be distributed along these buttresses. Therefore, a small impact force will result in a localized injury, whereas high impact trauma will result in more extensive fractures [5]. Tomich et al. showed orbital floor fracture as the most common injury following trauma in their series (18.3%) associated mainly with the maxillary sinus wall fracture [6]. This is similarly seen in a review by Alvi et al. in their study involving 151 patients with facial fractures. They found that most of MFT cases had orbital fracture involvement (24.2%) [1]. However, some studies indicated the mandibular fracture as the most common facial bone fracture [2, 3].

When dealing with trauma cases, patients must first be cleared of life-threatening conditions. The initial clinical assessment will determine if imaging is warranted. Thin-cut axial CT with coronal and sagittal reconstruction is usually the imaging of choice to evaluate both simple and complex orbital floor fractures. When a diagnosis has been confirmed by radiological assessment, the surgeon will then have to decide between operative intervention and conservative management. Urgent surgical repair is mandatory in patients presenting with unresolved oculocardiac reflexes (OCRs), commonly seen in pediatric patients with limited vertical eye movement due to the entrapped inferior rectus muscle or periorbital tissues evidently on imaging, a condition known as the “white-eyed” fracture [6]. Although an uncommon clinical finding, the OCR-associated orbital blowout fracture could lead to debilitating complications leading to bradycardia and even asystole.

Nonurgent repair is indicated even in patients with apparent enophthalmos causing functional or cosmesis concerns and in patients with diplopia on clinical ground [7]. Similarly, although extensive of more than 50%, both the size of the orbital floor fracture and its volume increment require intricate radiological assessment that commonly subjects patients for nonurgent repair [7]. In contrast to this, surgical treatment should not be delayed for cases with radiological and clinical evidence of muscle entrapment as this could potentially lead to fibrosis and rectus muscle ischemic necrosis associated with poor prognosis. In our case, the patient was subject to conservative management. Long-term follow-up that was carried out up for 6 months after trauma showed no enophthalmic complication.

It is worthwhile to note that the introduction of CBCT and its analysis in all radiographic planes have significantly improved the practice of many OMF surgeons, especially in making quick diagnosis and thorough preoperative assessment and surgical planning, if indicated. Its smaller size, lower cost, and easier operation have also made this modality rapidly accepted [8]. A study done by Kaeppler et al. showed that CBCT should be considered as the better alternative to MDCT for diagnosing mandibular fractures in ambulating patients without loss of consciousness [9]. Although CBCT can be successfully used for topographical bone imaging, it was suggested that it is not sufficient for soft tissue imaging and evaluation of the bone height [10]. Thus, the use of conventional CT, multidetector CT (MDCT), and MRI remains important. However, the use of conventional CT is limited in oral surgery practice due to high radiation dose, generation of image artifacts by metal-containing dental materials, and its cost and accessibility [10]. In recent years, MDCT has greatly aided in facial injury assessment, as this technology provides high-quality multiplanar views and enables 3D reconstruction. Ogura et al. believe that the use of MDCT remains the imaging tool of choice given the complexity of MFT, as it can concurrently detect maxillofacial fractures, the degree of fragment dislocation, soft tissue edema, and the presence of hemorrhage [11]. On the other hand, MRI is still superior in detailed visualization of soft tissue, including temporomandibular joint (TMJ) injuries, hemarthrosis, paranasal sinuses, and traumatic aneurysm [12]. Thus, in patients with MFT presenting with diplopia, MRI could serve as the imaging modality of choice to visualize herniation or entrapment of the inferior rectus muscle or periorbital tissues.

Despite subjecting a patient to conservative treatment after a maxillofacial trauma based on radiographic findings and interpretation, it is mandatory to follow up the patient's progress. Montezuma et al. reported 2 cases of progressive enophthalmos in nonmajor orbital and facial fracture patients [13]. The late enophthalmos developed when the orbital floor was retracted secondary to the contracted ipsilateral maxillary sinus. This phenomenon, which is known as a silent sinus syndrome (SSS), is characterized by progressive enophthalmos, hypoglobus, and pseudoptosis with deepening of the superior sulcus [14]. Following MFT, it is also thought that the direct damage to the osteomeatal complex (OMC) caused chronic osteomeatal outflow obstruction. Prolonged negative pressure will subsequently lead to the development of maxillary sinus atelectasis. Therefore, patients presenting with late enophthalmos with a background history of MFT should be subjected to careful radiological assessment of the OMC and other surrounding structures. Surgically restoring the patency of the OMC may be a part of the treatment plan especially in an impure orbital fracture. In addition, retrospective review by Choi et al. showed that late enophthalmos in an unoperated orbital fracture can be predicted via preoperative orbital volume ratio (OVR) measurement using CT [15], as seen in this case.
